# Take One!

**Published:** 1900-12

**Authors:** 


### TAKE ONE!

IN the flood of literature that is being turned upon medical journal readers on the subject of cocainization of the spinal cord, the following terms descriptive of the locality of injection have been noted: Spinal, intra-spinal, lumbar, intradural, subdural, medullary, intramedullary, rachidian, subarachnoid, subarachnoidal, interarachnoid, subarachnoidean, endomeningeal. And the list is apparently not yet complete.

---

WE desire to call especial attention to the following letter from Dr. Nicolson and to urge upon all who desire to attend the congress, with the intention of presenting papers on surgical subjects, to lose no time in notifying him of the fact and furnishing him with the title of the paper:

ATLANTA, GA., November 22, 1900.

DEAR DOCTOR:—As you are aware, the Third Pan-American Medical Congress will be held in Havana, Cuba, on the 26th, 27th, 28th and 29th of December next, and I write to ask if you cannot find time to contribute a paper to the Surgical Section upon any subject that you may desire. If you can do so, I would be pleased to have you send me, at the earliest convenient moment, the title of the same, that I may forward it to the General Secretary at Havana.

I earnestly hope that you may be able to present a paper, as we are anxious to have a good representation from the United States.

I should also be pleased if you can give me some idea as to the number of ladies who might possibly accompany your party.

Awaiting your early reply, and placing myself at your disposal for any further information in my power, I beg to remain, my dear doctor,

Yours very truly,

WILLIAM PERRIN NICOLSON.

Secretary of the Surgical Section for the U. S. Pan-American Congress.

